# Identification of an immunodominant peptide from citrullinated tenascin-C as a major target for autoantibodies in rheumatoid arthritis

**DOI:** 10.1136/annrheumdis-2015-208495

**Published:** 2015-12-09

**Authors:** Anja Schwenzer, Xia Jiang, Ted R Mikuls, Jeffrey B Payne, Harlan R Sayles, Anne-Marie Quirke, Benedikt M Kessler, Roman Fischer, Patrick J Venables, Karin Lundberg, Kim S Midwood

**Affiliations:** 1 Nuffield Department of Orthopaedics, Rheumatology and Musculoskeletal Sciences, Kennedy Institute of Rheumatology, University of Oxford, Oxford, UK; 2 Cardiovascular Epidemiology, Institute of Environmental Medicine, Karolinska Institutet, Stockholm, Stockholm, Sweden; 3 Department of Medicine, University of Nebraska, Medical Center, Omaha, Nebraska, USA; 4 Department of Surgical Specialties, University of Nebraska, Medical Center, College of Dentistry, Lincoln, Nebraska, USA; 5 Department of Biostatistics, University of Nebraska, Medical Center, Omaha, Nebraska, USA; 6 Nuffield Department of Medicine, Target Discovery Institute, University of Oxford, Oxford, UK; 7 Rheumatology Unit, Department of Medicine, Karolinska Institutet, Stockholm, Sweden

**Keywords:** Ant-CCP, Autoantibodies, Rheumatoid Arthritis

## Abstract

**Objectives:**

We investigated whether citrullinated tenascin-C (cTNC), an extracellular matrix protein expressed at high levels in the joints of patients with rheumatoid arthritis (RA), is a target for the autoantibodies in RA.

**Methods:**

Citrullinated sites were mapped by mass spectrometry in the fibrinogen-like globe (FBG) domain of tenascin-C treated with peptidylarginine deiminases (PAD) 2 and 4. Antibodies to cyclic peptides containing citrullinated sites were screened in sera from patients with RA by ELISA. Potential cross-reactivity with well-established anticitrullinated protein antibody (ACPA) epitopes was tested by inhibition assays. The autoantibody response to one immunodominant cTNC peptide was then analysed in 101 pre-RA sera (median 7 years before onset) and two large independent RA cohorts.

**Results:**

Nine arginine residues within FBG were citrullinated by PAD2 and PAD4. Two immunodominant peptides cTNC1 (VFLRRKNG-cit-ENFYQNW) and cTNC5 (EHSIQFAEMKL-cit-PSNF-cit-NLEG-cit-cit-KR) were identified. Antibodies to both showed limited cross-reactivity with ACPA epitopes from α-enolase, vimentin and fibrinogen, and no reactivity with citrullinated fibrinogen peptides sharing sequence homology with FBG. cTNC5 antibodies were detected in 18% of pre-RA sera, and in 47% of 1985 Swedish patients with RA and 51% of 287 North American patients with RA. The specificity was 98% compared with 160 healthy controls and 330 patients with osteoarthritis.

**Conclusions:**

There are multiple citrullination sites in the FBG domain of tenascin-C. Among these, one epitope is recognised by autoantibodies that are detected years before disease onset, and which may serve as a useful biomarker to identify ACPA-positive patients with high sensitivity and specificity in established disease.

## Introduction

Citrullination, the conversion of arginine residues to the non-standard amino acid citrulline, is catalysed by peptidylarginine deiminases (PAD). Levels of citrullinated proteins are significantly elevated at sites of inflammation including the joints of patients with rheumatoid arthritis (RA).^1^ ^2^ Whereas citrullination is ubiquitous in normal physiology and inflammation, anticitrullinated protein antibodies (ACPAs) are well established markers for the diagnosis of RA.^3^ ^4^ Appearing before evident symptoms, these autoantibodies correlate with poor prognosis and progressive joint destruction,^5–8^ and ACPA-positive patients often require more aggressive treatment.^9^

ACPAs are routinely detected using cyclic-citrullinated peptide (CCP) assays, designed to capture ACPA with maximum diagnostic sensitivity and specificity, using artificial peptides with no homology to naturally occurring proteins in the joint. While an excellent diagnostic test, these assays are of limited use in defining subsets of ACPA-positive patients and examining mechanisms of disease pathogenesis. At least 20 molecules recognised by ACPA have been described,^10^ but few of these have been demonstrated in the joint, epitope-mapped, antigen specificity confirmed and evaluated in independent large cohorts. Antigenic peptides described so far that fulfil all of these criteria include citrullinated fibrinogen (cFib),^11^ citrullinated vimentin (cVim),^12^ and citrullinated α-enolase peptide 1 (CEP-1).^13^ The diagnostic sensitivity of each of these peptides is relatively low, typically between 30% and 50%. However, when analysed in combination, sensitivity increases, and multiple serological subsets are demonstrated.^14^ Moreover, knowledge of the antigen specificity enables investigation of aetiological mechanisms. For example gene/environment (MHC shared epitope and smoking) interactions have been demonstrated with anti-CEP-1^15^ particularly when combined with dual positivity for anti-cVim.^14^ Knowledge of the antigens involved also reveals how ACPAs contribute to disease pathogenesis. For example, immune complexes containing cFib signal to induce proinflammatory cytokines,^16^ ^17^ and antibodies to cVim provoke osteoclastogenesis and bone erosion.^18^

Tenascin-C is a large, multimodular, extracellular matrix glycoprotein that is specifically upregulated during inflammation, but which is absent in most healthy tissues.^19^ ^20^ Tenascin-C levels are elevated in the cartilage, synovium and synovial fluid of patients with RA,^21–24^ as well as in RA serum where levels correlate with joint erosion.^25^ Tenascin-C stimulates inflammation; inducing de novo cytokine synthesis via activation of toll-like receptor 4 (TLR4),^26^ controlling cytokine synthesis post-transcriptionally via induction of microRNAs^27^ and regulating adaptive immunity by driving Th17 cell polarisation.^28^ ^29^ In murine models of arthritis tenascin-C expression is required to maintain chronic joint inflammation and the C-terminal fibrinogen-like globe domain (FBG) of tenascin-C is arthritogenic upon intra-articular injection.^19^

Tenascin-C can be found in immune complexes in the RA joint.^30^ Moreover, a citrullinated peptide from the FBG domain of tenascin-C was recently detected in RA synovial fluid.^31^ These data prompted us to investigate this arginine-rich domain of tenascin-C as a novel autoantigen. To characterise it with the criteria that have been applied to cFib, cVim and CEP-1, we epitope-mapped the FBG domain with a screening panel of RA sera, and examined the antigen specificity of two immunodominant epitopes by inhibition studies. We then went on to standardise an ELISA assay and used it to detect antibodies in pre-RA serum samples and two large independent patient cohorts with early and established RA.

## Material and methods

### Subjects

All RA cases fulfilled the 1987 American College of Rheumatology classification criteria.^32^ Four cohorts were examined, all from previously published studies with informed consent and ethical approval. (1) The screening cohort comprised 20 British patients with RA and 20 healthy individuals.^13^ (2) The 101 pre-RA cases and 326 matched controls were identified in a nested case-control study in four Southern European cohorts.^33^ (3) One thousand nine hundred and eighty-five cases of RA and 160 controls were from the Swedish population-based case-control study EIRA (Epidemiological Investigation of RA).^34^ Details of this EIRA cohort can be found in the online supplementary file. (4) Two hundred and eighty-seven patients with RA and 330 control patients with osteoarthritis (OA) were from the USA.^35^

### Citrullination reaction

Recombinant human FBG^26^ was incubated with rabbit skeletal muscle PAD2 (rPAD2), or recombinant human PAD2 or PAD4 (hPAD2, hPAD4), resolved on an SDS gel and stained with Coomassie-blue or western blotted with a monoclonal human antimodified citrulline (AMC) antibody (Modiquest Research, clone C4, 1:500). A detailed description of this method can be found in the online supplementary file.

### LC-MS/MS analyses

Citrullinated FBG was resolved on a 12% SDS gel and Coomassie-stained protein bands were excised and in gel-digestion performed as described.^36^ Peptides were analysed by LC-MS/MS. Details of this method can be found in the online supplementary file.

### Differential scanning fluorimetry and circular dichroism

Details can be found in the online supplementary file.

### Peptides, ELISAs and cross-reactivity assay

Details about peptides can be found in the online supplementary file. ELISAs were used to detect antibodies against citrullinated peptides in human sera as described.^13^ Briefly, 96-well plates were coated with 10 μg/mL peptide, blocked with 2% BSA and incubated with sera diluted 1:100. Bound antibodies were detected with an HRP conjugated antihuman IgGFc monoclonal antibody (Jackson—for EIRA study, Stratech—for all other ELISAs). A standard curve of positive sera was used to calculate relative antibody titres in arbitrary units (AU) for each sample. Subtraction of the OD450 of the native peptide from the OD450 of the citrullinated peptide was used to correct reactivity and dOD450 values were transformed into AU using the standard curve (dAU). The cross-reactivity assay is described in the online supplementary file.

### Statistical analysis and software

Details can be found in the online supplementary file.

## Results

### FBG is citrullinated in vitro by PAD2 and PAD4

**Figure 1:**
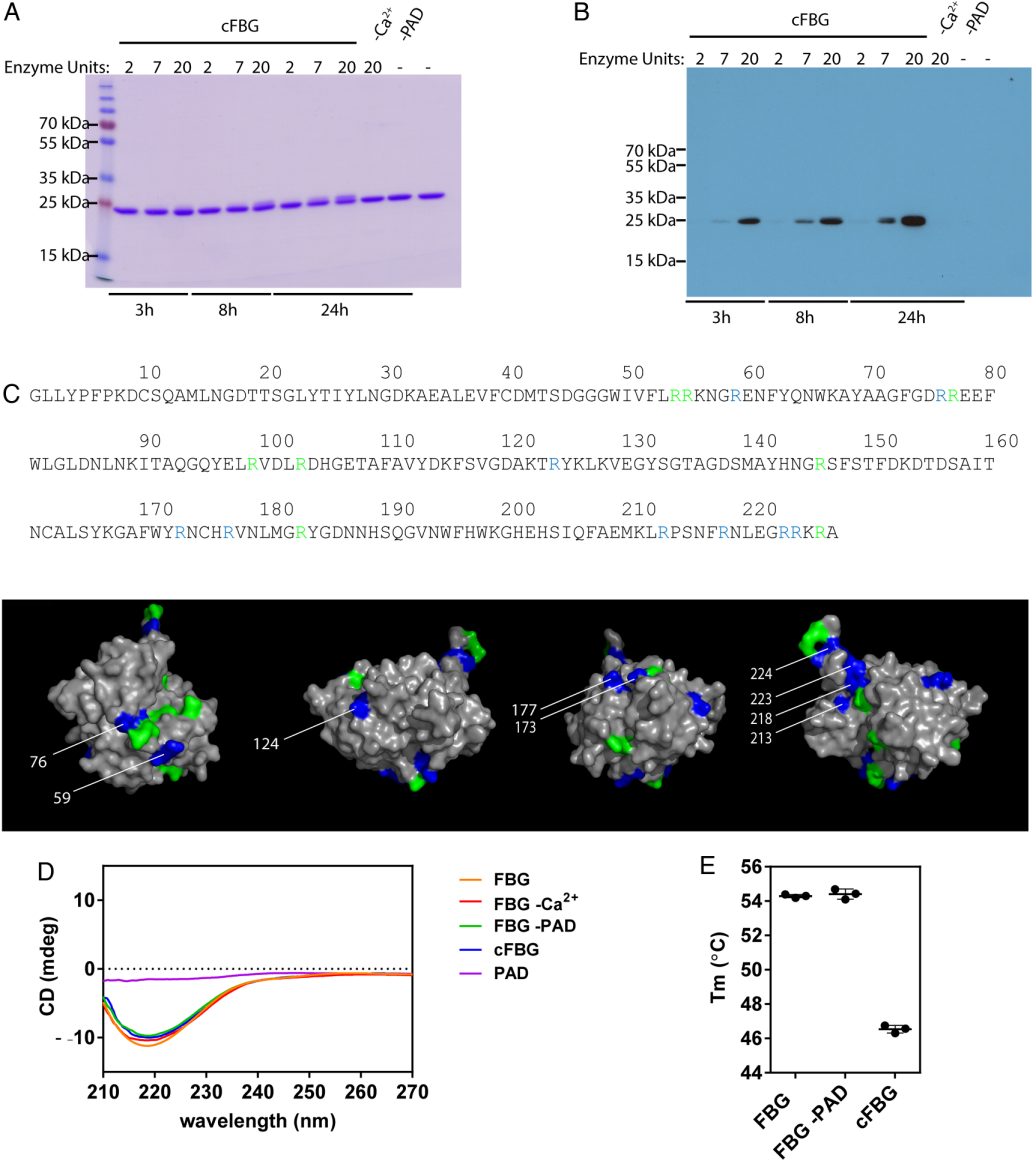
Citrullination of fibrinogen-like globe (FBG) by rPAD2. Coomassie stained SDS gels (A) and immunoblot probed with an antimodified citrulline (AMC) antibody (B) of FBG citrullinated by rPAD2. FBG incubated in buffer with rPAD2 but without Calcium (-Ca^2+^) or without enzyme (-PAD) served as negative controls. Untreated native FBG was loaded in the last well. (C) Arginine residues citrullinated by rPAD2, hPAD2 and hPAD4 were determined by LC-MS/MS. Arginine residues that were modified to citrulline are highlighted in blue, all non-citrullinated arginines are shown in green. (D) The super secondary structure of native and citrullinated FBG (3 h, 20U rPAD2/mg FBG) was analysed by circular dichroism. (E) The melting temperature of native and citrullinated FBG (3 h, 20U rPAD2/mg FBG) was analysed by differential scanning fluorimetry. Results are shown as mean ±SD from three independent experiments.

FBG was citrullinated by rPAD2, demonstrated by a small increase in the molecular weight on Coomassie-stained SDS-PAGE and western blotting with an AMC antibody (figure 1A,B). Mass spectrometry analysis of citrullinated FBG covered 14 of the 17 arginines present in this domain of tenascin-C, of which 9 were citrullinated (figure 1C, see online supplementary figure S1). rPAD2, hPAD2 and hPAD4 each citrullinated the same sites within FBG with no major difference in the degree of citrullination observed (see online supplementary figure S2). Circular dichroism showed comparable spectra between native FBG and citrullinated FBG (figure 1D) indicating that citrullination of FBG does not impact the secondary structure of the protein. Differential scanning fluorimetry however revealed a significantly lower melting temperature of citrullinated FBG (46.5±0.2) compared with FBG (54.3±0.1) (figure 1E), demonstrating that citrullination leads to partial protein unfolding.

### cTNC1 and cTNC5 are the primary epitopes recognised by ACPA in patients with RA

**Figure 2:**
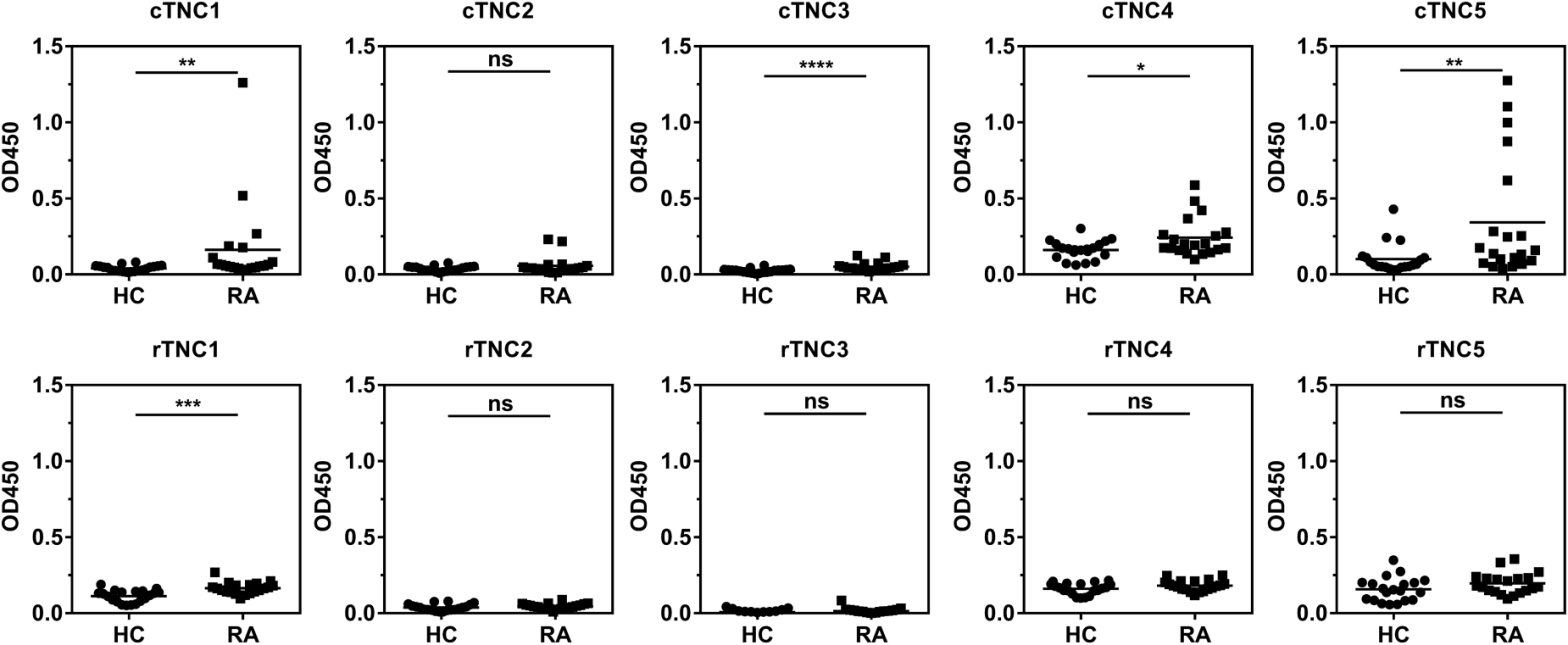
Identifying the citrullinated antibody epitope. IgG response to citrullinated fibrinogen-like globe (FBG) peptides (cTNC) and arginine containing control peptides (rTNC) in patients with rheumatoid arthritis (RA; n=20) and healthy controls (HC, n=20). Mann-Whitney U test was used to calculate p values for differences between groups (ns=no significant difference, *p<0.05 and **p<0.01, ***p<0.001, ****p<0.001). cTNC, citrullinated tenascin-C.

Five tenascin-C cyclic peptides encompassing the citrullinated residues identified by mass-spectrometry, together with their arginine-containing controls (see online supplementary table S1), were used to map antibody response in a screening panel of serum samples from 20 patients with RA, and from 20 healthy subjects, by ELISA. Antibodies to citrullinated tenascin-C (cTNC) peptides cTNC1 and cTNC5 were detected in serum samples from 35% and 40% of patients, respectively, but not in control sera, with no response against the arginine-containing control peptides (rTNC). There was little or no reactivity with the other three peptides tested (figure 2). Therefore cTNC1 and cTNC5 were selected for further study.

### Anti-cTNC antibodies show limited cross-reactivity with other ACPA epitopes

**Figure 3:**
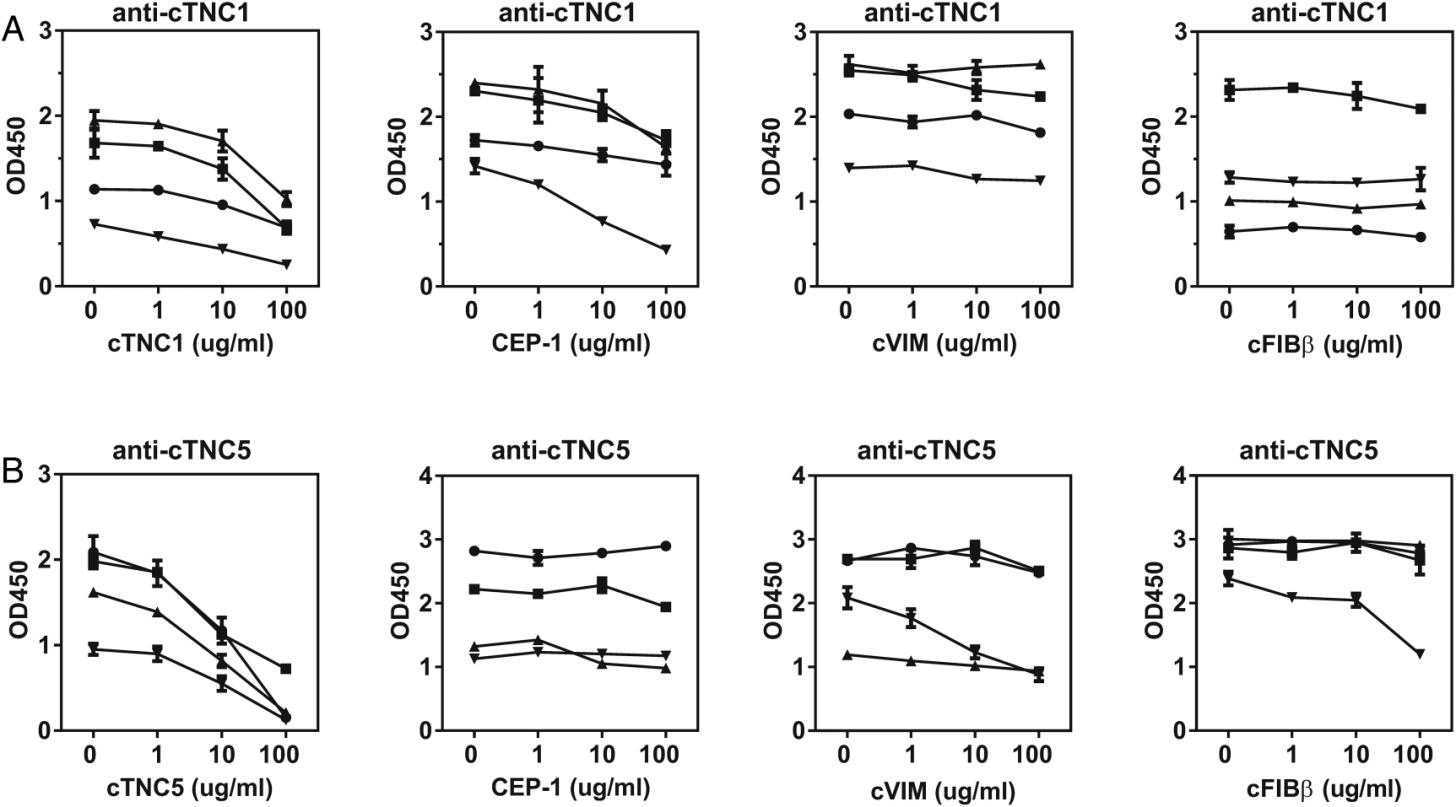
Anti-cTNC antibody cross-reactivity with CEP-1, cVIM and cFIBβ. Sera, double-reactive with peptides cTNC1 or cTNC5 and CEP-1, cVIM or cFIBβ, respectively, were preincubated with increasing concentrations of the indicated peptides, and the IgG response to cTNC1 (A) and cTNC5 (B) was measured. CEP-1, citrullinated α-enolase peptide 1; cFib, citrullinated fibrinogen; cTNC, citrullinated tenascin-C; cVim, citrullinated vimentin.

To examine epitope specificity and potential cross-reactivity of anti-cTNC antibodies with already identified ACPA antigens, inhibition experiments were performed with the well defined peptides of CEP-1 (^5^KIHA-cit-EIFDS-cit-GNPTVE^21^), cVIM (^59^VYAT-cit-SSAV-cit-L-cit-SSVP^74^) and cFIBβ (^36^NEEGFFSA-cit-GHRPLDKK^52^). Absorption by the homologous peptides was more efficient for cTNC5 than cTNC1. There was no cross-reactivity between anti-cTNC1 and cVIM and cFIBβ, though there was some inhibition by the CEP-1 peptide (17–70% inhibition) (figure 3A). In contrast, there was no cross-reactivity between anti-cTNC5 and CEP-1, while these antibodies showed limited cross-reactivity with cVIM and cFIBβ in one serum sample (inhibition by 58% and 50%, respectively) (figure 3B).

**Figure 4:**
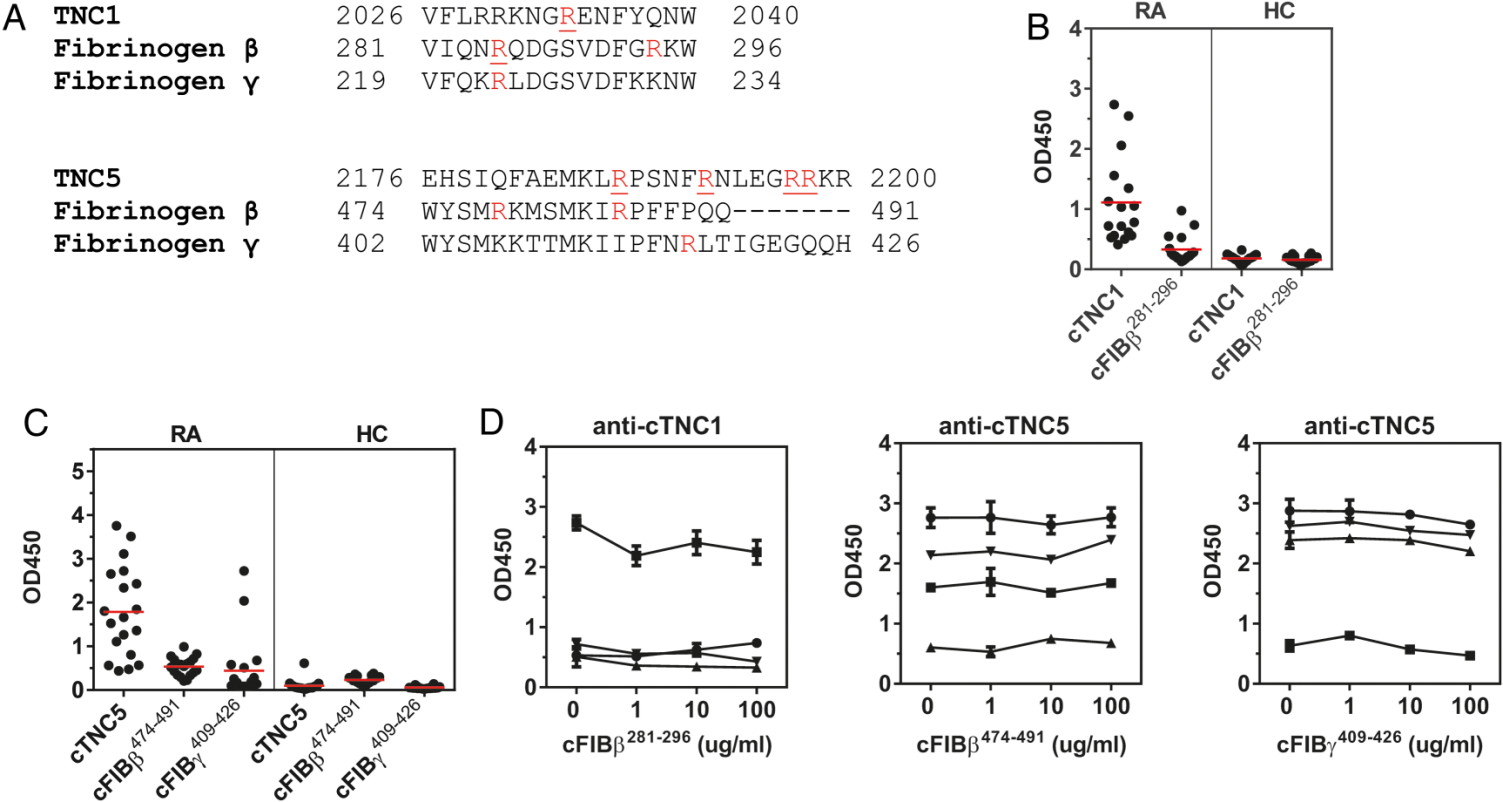
Anti-cTNC antibody cross-reactivity with homologous fibrinogen peptides. (A) Multiple sequence alignment (Clustal Omega) of tenascin-C, fibrinogen β chain and fibrinogen γ chain. Arginines found citrullinated in vitro are highlighted in red, citrullinated arginines described as anticitrullinated protein antibody (ACPA) epitopes are underlined. (B and C) IgG response to cTNC1, cTNC5 and homologous cFib peptides was measured in sera positive for cTNC1 (B) or cTNC5 (C). (D) Sera, double-reactive to peptides cTNC1 or cTNC5 and the homologous cFib peptides were preincubated with increasing concentrations of the indicated peptides, and IgG response to cTNC1 and cTNC5 was measured. cFib, citrullinated fibrinogen; cTNC, citrullinated tenascin-C; HC, healthy controls; RA, rheumatoid arthritis.

Because the FBG domain of tenascin-C exhibits some sequence homology with fibrinogen we also analysed whether anti-cTNC antibodies cross-react with epitopes on citrullinated peptides containing similar sequences of fibrinogen β chain (cFibβ^281–296^) and fibrinogen γ chain (cFibβ^474–491^, cFibγ^409–426^) (figure 4A, for peptide sequences see online supplementary table S2). From 17 sera reactive with cTNC1, 7 also reacted with cFibβ^281–296^ (figure 4B), from 19 sera reactive with cTNC5, 14 also reacted with cFibβ^474–491^, and 9 with cFibγ^409–426^ (figure 4C). To examine whether this dual positivity was due to true cross-reactivity, four samples that were double positive for cTNC and cFib peptide IgG were tested by inhibition experiments. No reduced reactivity to cFBG epitopes was observed when sera were preincubated with cFibβ and cFibγ peptides (figure 4D).

### Anti-cTNC5 is detected in pre-RA sera and with moderate-to-high sensitivity in early and established RA

**Figure 5:**
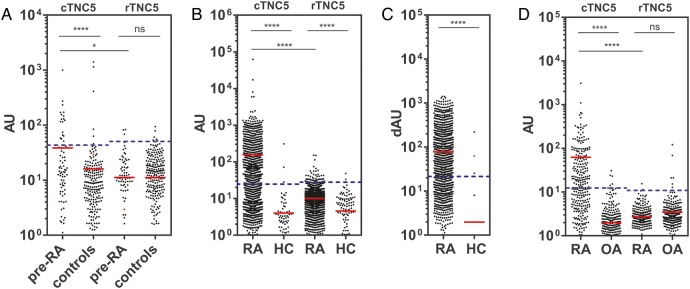
Anti-cTNC5 antibody response in rheumatoid arthritis (RA) and pre-RA sera. IgG response to cTNC5 and rTNC5 in serum samples from (A) 101 pre-RA individuals (pre-RA) and 326 controls, and (B) from 1985 patients with RA (RA) and 150 healthy controls (HC) from the Epidemiological Investigation of RA (EIRA) cohort. (C) IgG response to cTNC5 in the EIRA cohort in dAU when controlled for binding to arginine control peptide. dAU was calculated as described in Material and Methods. (D) IgG response to cTNC5 and rTNC5 in 287 serum samples from patients with RA (RA) and 330 serum samples from patients with osteoarthritis (OA) from a US cohort. The red line indicates the mean. Blue dotted lines indicate cut-off for positivity. Mann-Whitney U test was used to calculate p values for differences between groups (ns=no significant difference, *p<0.05, ****p<0.001). AU, arbitrary units; cTNC, citrullinated tenascin-C.

In 101 pre-RA sera (median 7 years before diagnosis), 18% of pre-RA sera were positive for anti-cTNC5 antibodies (figure 5A) compared with 2% of 326 sera from controls. No antibodies against cTNC1 were detected (data not shown). Therefore, because cTNC5 appeared to have better antigen specificity in our absorption experiments and a higher frequency of antibodies in pre-RA and established RA, further analysis focused on cTNC5.

In the EIRA cohort, 47% of 1985 RA sera and 2% of 160 healthy control sera were positive for antibodies to cTNC5, indicating a diagnostic sensitivity of 47% and specificity of 98%. Within the RA sera, 2.5% also bound the arginine-control peptide rTNC5 (figure 5B), and when controlling for binding to the arginine control peptide the sensitivity remained moderately high at 41% (figure 5C). This figure was higher than the frequency of citrulline-specific antibodies to CEP-1 (35%), anti-cVIM (37%) and anti- cFIBβ (37%), measured in the same cohort by ELISA.^14^

We confirmed the moderate-to-high diagnostic sensitivity of cTNC5 (51%) in an independent US cohort of 287 sera from patients with RA and 330 sera from OA disease controls (figure 5D). In this cohort the binding to rTNC5 was not increased compared with OA.

**Figure 6:**
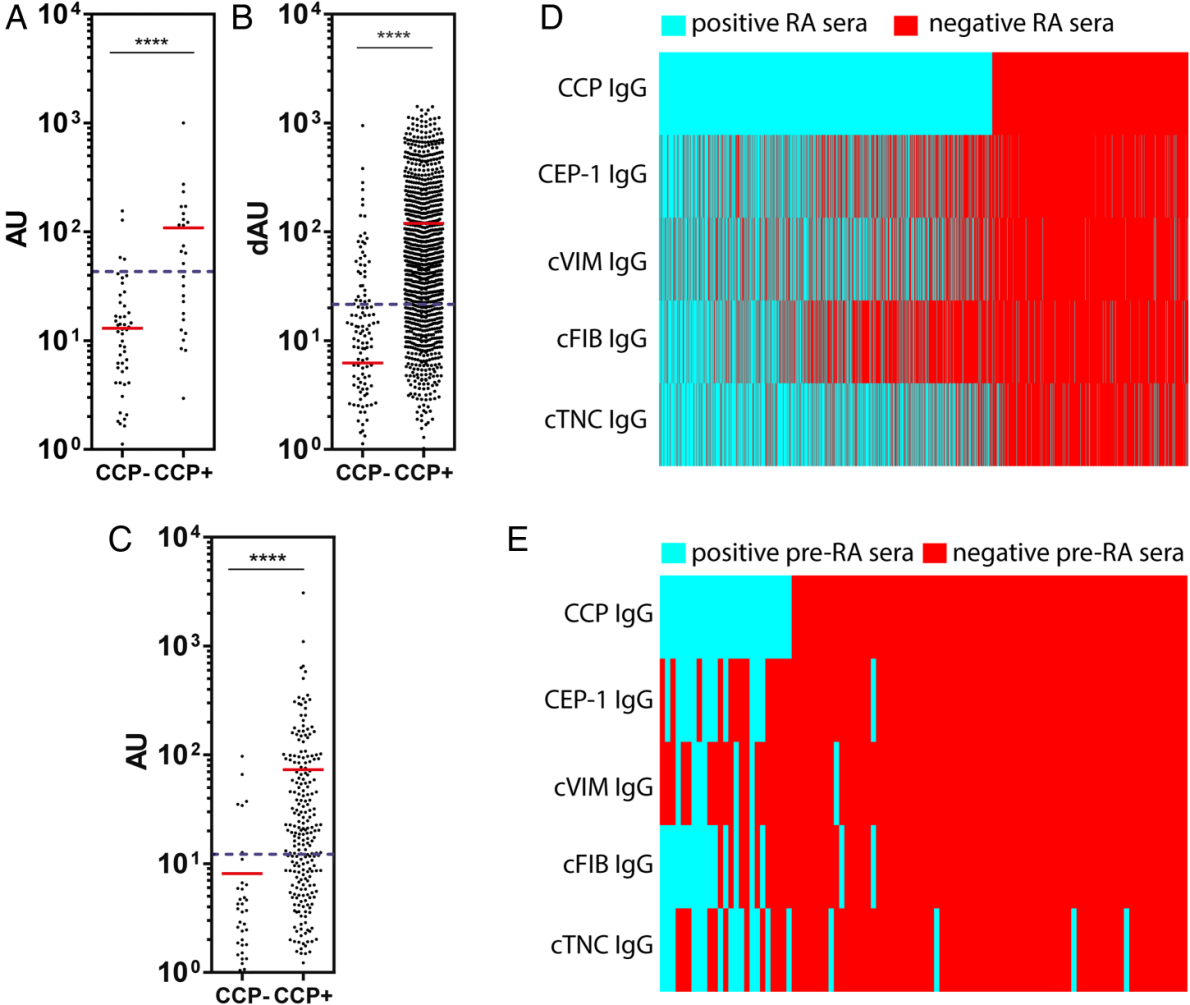
Anti-cTNC antibody levels correlate with cyclic-citrullinated peptide (CCP2) levels. IgG response to cTNC5 in (A) CCP2-positive (CCP2+, n=1255) and CCP2-negative (CCP2−, n=730) sera of patients with rheumatoid arthritis (RA) of the Epidemiological Investigation of RA (EIRA) cohort, (B) CCP2-positive (n=240) and CCP2-negative (n=47) sera of patients with RA of the US cohort and (C) in CCP2-positive (n=27) and CCP2-negative (n=74) sera of pre-RA individuals. (D and E) Heat maps showing the presence (blue) or absence (red) of an IgG antibody response to anticitrullinated protein antibody (ACPA) epitopes in RA sera from the EIRA cohort (D) or pre-RA sera (E). cTNC, citrullinated tenascin-C; RA, rheumatoid arthritis.

Anti-cTNC5 reactivity was significantly higher in CCP2-positive patients compared with CCP2-negative patients in RA (figure 6A,B) and pre-RA samples (figure 6C). Anti-cTNC5 antibody largely overlapped with other ACPA (figure 6D,E, see online supplementary figure S3) in the RA and pre-RA cohorts. However in the EIRA cohort 5.4% of the serum samples exclusively reacted with the cTNC5 peptide alone. In the anti-CCP2 negative samples 4.9% reacted with cTN5.

In EIRA cTNC5-positive RA was associated with smoking (OR 1.65 vs 1.26) and *HLA-DRB1* shared epitopes (OR 4.98 vs 1.68), but not with *PTPN22* (OR 1.77 vs 1.44) when compared with the cTNC5-positive/CCP2-negative RA subset (see online supplementary table S3). We also analysed whether cTNC5 antibodies are associated with specific HLA-DRB1 epitopes and found that cTNC5 antibodies did not associate with DRB1*10 alleles, but with HLA-DRB1*01 and DRB1*04 alleles (see online supplementary table S4). Antibodies against cTNC5 negatively associated with HLA-DR13 (see online supplementary table S5).

In the US cohort, cTNC5 antibody positivity was significantly associated with disease activity (DAS 28-CRP), but did not associate with other analysed clinical parameters (disease duration, swollen and tender joints, sharp score and erosion score) (see online supplementary table S6).

## Discussion

In this study, we describe a novel citrullinated peptide from the FBG domain of tenascin-C. The citrullinated residues can be generated by either PAD2 or PAD4, yielding epitopes that are recognised by antibodies in approximately one of every five individuals with preclinical RA and with a moderate-to-high diagnostic sensitivity in early and established disease. Inhibition assays and analysis of antibodies to other well characterised peptides indicate that anti-cTNC5 antibody status is independent of reactivity to other citrullinated peptides. Even though a large number of antigenic citrullinated peptides have been described as reactive with ACPA in previous reports, few have been examined with the stringent criteria used in this study. Therefore, our findings suggest that cTNC5 is a novel and independent addition to the relatively small number of citrullinated peptides which are genuinely targeted by ACPA, and which may have a role in clinical diagnosis and investigating pathogenesis in RA.

The FBG domain of tenascin-C was citrullinated in vitro by PAD2 and PAD4. While these enzymes have different substrate specificities,^37^ both modified the same nine arginines in FBG to a similar degree. Lack of citrullination of five other arginines in FBG by any PAD reflects the specificity of this modification, likely due to hindered accessibility of these residues, or unfavourable neighbouring amino acids. Citrullinated arginines were located at five distinct sites within FBG, of which two, cTNC1 and cTNC5, were reactive with sera from patients with RA. However, antibodies to only one, cTNC5, were detected in sera of pre-RA cases.

The different associations of cTNC1 and cTNC5 suggest that cTNC5 may be important in priming the ACPA response, whereas antibodies to cTNC1 may arise as a result of epitope spreading in more established disease. These data also reflect that the autoantibody response in RA is not citrulline-specific; instead it depends on the whole epitope around the modified residue including neighbouring amino acids and the three-dimensional structure.^10^ It is well documented that distinct ACPA responses to different citrullinated epitopes within one protein exist, as described for example for citrullinated α-enolase^13^ or citrullinated fibrinogen.^38^ The peptide sequence of cTNC5 is predicted to form a very distinct, exposed structure at the very C-terminus of tenascin-C, potentially rendering it more easily accessible to ACPA than cTNC1. In addition, four sites are citrullinated within TNC5, compared with only a single citrullinated site within cTNC1, which may also contribute to the higher frequency of cTNC5 ACPA observed.

The frequency of anti-cTNC5 antibodies in the pre-RA cohort (18%) is comparable to antibody frequencies described for other ACPAs in the same cohort, including cFIBβ (18%) and CEP-1 (15%).^33^ Analysis of a large cohort of patients with early RA demonstrated moderate-to-high sensitivity of RA samples for cTNC5 (47%). This is the highest recorded sensitivity for any single antigenic peptide in this cohort, in this case compared with a 35–37% sensitivity for antibodies to each of the three other antigenic peptides.^14^ We also found reactivity to cTNC5 at a similar frequency (51%) in a second cohort of RA sera from US patients.

ACPAs generally show limited cross-reactivity.^14^ ^39^ In line with these reports, we showed that antibodies to cTNC exhibited little cross-reactivity with cFib, cVim and CEP-1, and are distinct from antibodies reacting with peptides from homologous regions in fibrinogen. cTNC5 ACPA-positive sera were mostly found within the anti-CCP2 antibody-positive RA population with cTNC5 antibody levels highest in the anti-CCP2 antibody positive subgroup, as described for other ACPA.^14^ Of the patients with RA 4.9% within the anti-CCP2 antibody-negative group were also anti-cTNC5 ACPA-positive, demonstrating that not all ACPA-positive patients can be detected by testing for CCP reactivity. Moreover, a subset of CCP-positive patients with RA was single-positive for cTNC5 antibodies (5.4%), revealing cTNC5 as a distinct ACPA fine specificity in RA sera and indicating that assaying this ACPA alone would be helpful in diagnosing patients that might otherwise be missed. Combined testing for several specific ACPAs has been shown to increase diagnostic sensitivity and specificity.^40^ Together these data suggest that the addition of cTNC5 to an assay combining multiple ACPAs, as well as analysis of anti-cTNC5 alone, might be beneficial approaches in diagnosing RA.

*HLA-DRB1* SE alleles are associated with ACPA-positive RA.^41^ We found a strong association of anti-cTNC5 antibodies with SE positivity, as has been described for antibodies to other citrullinated antigens, like CEP-1 and cVIM.^14^ ^15^ cTNC5 antibodies mainly associated with HLA-DRB1 subtypes DRB1*04 as described for other ACPAs.^15^ ^39^ HLA-DR13 alleles protect against ACPA-positive RA,^42^ and we show here that it is also protective against cTNC5-positive RA. However, we did not observe a statistical significant association of *PTPN22,* another genetic risk factor for RA,^43^ with anti-cTNC5-positive RA. Smoking is a well established environmental risk factor for ACPA-positive RA^34^ ^44^ and here we describe a positive association of cTNC5-positive RA with smoking in the EIRA cohort, similarly as it has been described for antibodies against CEP-1 and cVIM.^14^ ^15^ Smoking-induced inflammation, in the context of chronic obstructive pulmonary disease (COPD), is associated with enhanced citrullination and may contribute to the generation of ACPA.^45^ ^46^ Interestingly, high tenascin-C expression was detected in lungs of patients with COPD compared with non-smokers.^47^ ^48^ Another risk factor for RA, is periodontitis.^49^ *Porphyromonas gingivalis* is a major periodontal pathogen and possesses a unique bacterial PAD enzyme which citrullinates bacterial and endogenous host peptides.^50^ ^51^ Tenascin-C is also expressed in periodontal tissue, and tenascin-C fragments were detected in gingival crevicular fluid of patients with periodontitis.^52^ Our results and these studies therefore reveal potential mechanisms for the generation of antigenic cTNC peptides in RA.

ACPAs are produced locally within the RA joint and may contribute directly to disease pathogenesis.^53^ For example immune complexes containing cFib stimulate cytokine synthesis in macrophages via activation of Fcγ-receptor and TLR4^17^ and, due to the homology of fibrinogen and the FBG domain of tenascin-C, it is conceivable that immune complexes containing cTNC may contribute to disease pathogenesis through a similar mechanism. Furthermore, citrullinated proteins themselves can be pathogenic, as described for cFib.^16^ ^17^ ^54^ It will be interesting to see if ACPA for cTNC5 bind to cTNC found within the RA joint and trigger cytokine formation in the form of immune complexes, or whether citrullination of the FBG domain enhances its activation of TLR4.^26^ The citrullinated FBG peptide previously detected in RA synovial fluid^31^ comprised the sequence we found in cTNC1. However, further citrullinated sites and ACPA epitopes are likely to be found in other domains of tenascin-C, as for example in the fibronectin type-III like repeats that share sequence homology with fibronectin, a molecule also found in synovial fluid and which is targeted by the autoimmune response in RA.^55^

The CCP-positive subset of patients with RA is linked with a more severe disease development and worse prognosis.^5^ ^7^ ^8^ However, no association of specific ACPAs with clinical parameters has been described so far.^56^ Here, we found that cTNC5 antibodies do not correlate with a number of clinical parameters, however, there was a significant association of cTNC5 antibodies with disease activity (DAS28-CRP), suggesting that cTNC5 antibodies may be a useful tool for predicting clinical outcome.

In conclusion, we describe an immunodominant peptide from tenascin-C, which is distinct from the other major antigenic citrullinated peptides described to date, and at least equal if not superior in terms of diagnostic sensitivity and specificity when used as an antigen in ELISA. Furthermore, previous demonstrations of the proinflammatory effects of tenascin-C and its detection at the site of inflammation suggest that immune responses to the FBG domain may be important in the aetiology and pathogenesis of RA.

## Supplementary Material

Web supplement
