# Supplementary material for: Identification of an immunodominant peptide from citrullinated tenascin-C as a major target for autoantibodies in rheumatoid arthritis
Source: Ann Rheum Dis. 2015 Dec 9;75(10):1876–83. doi: 10.1136/annrheumdis-2015-208495 (PMC5036245; doi:10.1136/annrheumdis-2015-208495)

Supplementary Information

Identification of an immunodominant peptide from citrullinated tenascin-C as a major target for autoantibodies in rheumatoid arthritis

Anja Schwenzer, Xia Jiang, Ted R. Mikuls, Jeffrey B. Payne, Harlan R. Sayles, Anne-Marie Quirke, Benedikt M. Kessler, Roman Fischer, Patrick J. Venables, Karin Lundberg & Kim S. Midwood

Supplementary Methods:

EIRA cohort

EIRA controls were randomly selected from the population registry, to match EIRA cases on age-, gender- and residential area[1]. Smoking data was collected by questionnaire at baseline. Subjects were categorized as ever-smokers or never-smokers[1]. *HLA-DRB1* subtyping and genotyping of the protein tyrosine phosphatase gene (*PTPN22* rs2476601) was described before[2,3].

Citrullination reaction

Recombinant human FBG[4] was incubated with rabbit skeletal muscle PAD2 (rPAD2) (Sigma Aldrich), or recombinant human PAD2 or PAD4 (hPAD2, hPAD4) (Modiquest Research) in citrullination buffer (50 mM Tris pH 8, 150 mM NaCl, 10 mM $CaCl_2$) with 5 mM DTT for 3, 8 or 24 hours at 37 ˚C. 1 µg of each sample was resolved on a 12% SDS gel and stained with Coomassie-blue or Western blotted with a monoclonal human anti-modified citrulline (AMC) antibody (Modiquest Research, clone C4, 1:500), following chemical modification of the membrane (0.0125% $FeCl_3$, 2.3 M H_2SO_4, 1.52 M H_3PO_4, 0.25 M Acetic Acid, 0.25% 2, 3-butanedione monoxime, 0.125% antipyrine).

LC-MS/MS analyses

Peptides were analysed on a LC-MS/MS workflow comprising a Dionex Ultimate 3000 nLC system coupled to a Q-Exactive mass spectrometer (Thermo Scientific) [5,6]. Briefly, chromatographic separation was achieved using a 50cm nEASY spray column (Thermo Scientific, (PepMAP C18, 75 µm x 500mm, 2 µm particle size) and a linear acetonitrile gradient from 2-35% in 5% DMSO and 0.1% formic acid. Precursor peptides were detected with a resolution of 70000 at 200m/z followed by the selection of up to 15 precursor ions. Raw data was imported into Progenesis QI (Waters, V4.1.4832.42146) for label-free quantitation and alignment, and peptides were identified with PEAKS (Bioinformatics Solutions, V7). The abundance of citrullinated peptides was normalised to the abundance of their non-citrullinated counterparts, which value was further normalised to the citrullinated peptide abundance detected in a non-citrullinated FBG control sample.

Differential Scanning Fluorimetry and Circular Dichroism

Five µM FBG was mixed with 5x SYPRO® orange protein gel stain (Life Technologies) in citrullination buffer and melting curves from 38 ˚C up to 99 ˚C recorded on the Applied Biosystems ViiA 7 Real-Time PCR System (excitation 550 nm, emission, 586 nm). The melting temperature (Tm) of each sample was defined as the maximum of the first derivate of the fluorescence. Circular dichroism spectra of FBG samples (200 µg/ml) were recorded using a Jasco J-815 CD Spectrometer, between 210 and 270 nm.

Peptides

Peptides from tenascin-C (accession number P24821) and fibrinogen β (P02675) and γ (P02679) chains (Table S1 and S2) were synthesized at a purity > 90-95%, with C- and N-terminal cysteines (Pepceuticals) and solubilized in water at 10 mg/ml. CEP-1, cVIM and cFIBβ peptides used for cross-reactivity assays are described elsewhere[7].

Crossreactivity ELISA

Cross-reactivity was analysed in human sera that were reactive to both cTNC1 or cTNC5 and CEP-1, cVIM, cFIBβ or citrullinated homologous fibrinogen peptides. Sera were diluted 1:100, incubated with 1, 10 and 100 µg/ml of peptides for 2 hours, centrifuged at 10,000 g for 10 min and the supernatant added to peptide-coated plates for analysis by ELISA as described in Material and Methods.

Statistical Analysis and Software

Mann–Whitney U tests were used to calculate p values for differences between groups (GraphPad Prism). The 98% percentile of healthy control samples was used to define the cut-off for positive antibody levels. Chi-square tests were used to compare frequency distributions of categorical variables while t-tests and Wilcoxon rank-sum tests were used to compare all continuous variables. To determine the association of smoking, *HLA-DRB1* SE (subtypes *DRB1**01 (except *DRB1**0103), *DRB1**04 and *DRB1**10), HLA-*DRB1**13 and *PTPN22* with different RA subsets, odds ratios (OR) with 95% Confidence Intervals (95% CI) were calculated using unconditional logistic regression models, with unexposed cases and controls as reference group. Analyses were adjusted for age, gender and residential area. All analyses were performed using SAS version 9.3. Heatmaps were generated using G-ProX and three-dimensional models were created using Pymol (Schrödinger, LLC).

1. Stolt P, Bengtsson C, Nordmark B, et al. Quantification of the influence of cigarette smoking on rheumatoid arthritis: results from a population based case-control study, using incident cases. *Ann Rheum Dis*. 2003;62:835-41.
2. Kallberg H, Padyukov L, Plenge RM, et al. Gene-gene and gene-environment interactions involving HLA-DRB1, PTPN22, and smoking in two subsets of rheumatoid arthritis. *Am J Hum Genet*. 2007;80:867-75.
3. Padyukov L, Silva C, Stolt P, et al. A gene-environment interaction between smoking and shared epitope genes in HLA-DR provides a high risk of seropositive rheumatoid arthritis. *Arthritis Rheum*. 2004;50:3085-92.
4. Midwood K, Sacre S, Piccinini AM, et al. Tenascin-C is an endogenous activator of Toll-like receptor 4 that is essential for maintaining inflammation in arthritic joint disease. *Nat Med*. 2009;15:774-80.
5. Fischer R, Kessler BM. Gel-aided sample preparation (GASP)--a simplified method for gel-assisted proteomic sample generation from protein extracts and intact cells. *Proteomics*. 2015;15:1224-9.
6. Montgomery AB, Kopec J, Shrestha L, et al. Crystal structure of Porphyromonas gingivalis peptidylarginine deiminase: implications for autoimmunity in rheumatoid arthritis. *Ann Rheum Dis*. 2015.
7. Cartwright AJ, Quirke A-M, de Pablo P, et al. Smoking Affects Immune Response to Citrullinated Autoantigens Before Clinical Onset of Rheumatoid Arthritis in a Southern European Cohort [abstract]. *Rheumatology (Oxford)*. 2014;53:i36.

Supplementary Table 1. Sequences of FBG peptides used for ELISAs.

FBG peptides	Sequence
rTNC1 (aa 2026-2040)	CVFLRRKNG-R-ENFYQNWC
cTNC1	CVFLRRKNG-cit-ENFYQNWC
rTNC2 (aa 2042-2058)	CAYAAGFGD-R-REEFWLGLC
cTNC2	CAYAAGFGD-cit-REEFWLGLC
rTNC3 (aa 2091-2106)	CFSVGDAKT-R-YKLKVEGYC
cTNC3	CFSVGDAKT-cit-YKLKVEGYC
rTNC4 (aa 2141-2157)	CKGAFWY-R-NCH-R-VNLMGRC
cTNC4	CKGAFWY-cit-NCH-cit-VNLMGRC
rTNC5 (aa 2183-2200)	CEMKL-R-PSNF-R-NLEG-R-R-KRC
cTNC5	CEMKL-cit-PSNF-cit-NLEG-cit-cit-KRC

Supplementary Table 2. Fibrinogen peptides homologous to citrullinated FBG peptides.

Fibrinogen peptides	Sequence
**cFIBβ$^{281\text{-}296}$**	CVIQN-cit-QDGSVDFG-cit-KWC
**cFIBβ$^{474\text{-}491}$**	CWYSMRKMSMKI-cit-PFFPQQC
**cFIBγ$^{409\text{-}426}$**	CTMKIIPFN-cit-LTIGEGQQHC

Supplementary Table 3. Association between smoking, any SE, *PTPN22* and different RA subgroups (in EIRA).

Subgroup	Exposure		OR (95%CI)	p-value (+/+ vs +/-)
	**Never smokers**	**Ever smokers**		
**Controls**	2114(43.46)	2750(56.54)	1.0 ref.	
**CCP2/cTNC5**				
**-/-**	85(41.87)	118(58.13)	0.86 (0.64-1.17)	
**-/+**	12(30.00)	28(70.00)	1.45 (0.72-2.93)	
**+/-**	201(33.84)	393(66.16)	1.26 (1.03-1.53)	
**+/+**	309(27.01)	835(72.99)	1.65 (1.41-1.93)	0.0049
	**None SE**	**Any SE**		
**Controls**	959(49.87)	964(50.13)	1.0 ref.	
**CCP2/cTNC5**				
**-/-**	94(46.31)	108(53.20)	1.15 (0.85-1.56)	
**-/+**	15(35.71)	27(64.29)	1.75 (0.91-3.34)	
**+/-**	218(36.64)	368(61.85)	1.68 (1.37-2.06)	
**+/+**	191(16.68)	941(82.18)	4.98 (4.11-6.04)	<0.0001
	**None PTPN22**	**Any PTPN22**		
**Controls**	1533(79.18)	403(20.82)	1.0 ref.	
**CCP2/cTNC5**				
**-/-**	126(62.07)	50(24.63)	1.65 (1.13-2.41)	
**-/+**	24(57.14)	12(28.57)	2.15 (1.03-4.49)	
**+/-**	371(62.35)	136(22.86)	1.44 (1.12-1.86)	
**+/+**	682(59.56)	314(27.42)	1.77 (1.44-2.18)	0.08

Supplementary Table 4. Associations between DR*01, *04 and *10 and different RA subgroups with the combination of presence or absence of CCP and cTNC5. OR were adjusted for age, gender, residential area, smoking, alcohol consumption and other SE. NA – not analysed.

Subgroup	Exposure		OR**(95%CI)	p-value (+/+ vs +/-)
	None	Any		
**DR*01**				
Controls	375(59.43)	256(40.57)	1.0ref.	
CCP2/cTNC5				
-/-	62(30.54)	45(22.17)	0.91(0.40-2.11)	
-/+	17(40.48)	10(23.81)	NA	
+/-	238(40.00)	129(21.68)	1.00(0.62-1.63)	
+/+	627(54.76)	310(27.07)	1.81(1.26-2.60)	0.01
**DR*04**				
Controls	220(34.87)	411(65.13)	1.0ref.	
CCP2/cTNC5				
-/-	41(20.20)	66(32.51)	0.77(0.33-1.84)	
-/+	13(30.95)	14(33.33)	NA	
+/-	110(18.49)	257(43.19)	1.29(0.78-2.15)	
+/+	192(16.77)	745(65.07)	3.46(2.35-5.11)	< 0.0001
**DR*10**				
Controls	609(96.51)	22(3.49)	1.0ref.	
CCP2/cTNC5				
-/-	102(50.25)	5(2.46)	1.18(0.36-3.87)	
-/+	24(57.14)	3(7.14)	NA	
+/-	351(58.99)	16(2.69)	1.53(0.71-3.29)	
+/+	898(78.43)	39(3.41)	2.11(1.15-3.88)	0.13

Supplementary Table 5. Association between DR13 and different RA subgroups with the combination of presence or absence of CCP and cTNC5. OR were adjusted for age, gender, residential area, smoking, alcohol consumption and any SE.

Subgroup	Exposure		OR (95%CI)	p-value
	**No DR13**	**Any DR13**		
**Controls**	503(79.71)	128(20.29)	1.0ref.	
**CCP2/cTNC5**				
**-/-**	86(42.36)	21(10.34)	0.84(0.49-1.46)	
**-/+**	25(59.52)	2(4.76)	0.31(0.07-1.34)	(-/+ vs -/-) 0.16
**+/-**	315(52.94)	52(8.74)	0.67(0.47-0.97)	
**+/+**	867(75.72)	70(6.11)	0.30(0.22-0.42)	(+/+ vs +/-) 0.0003

Supplementary Table 6. Association of cTNC5 antibodies with RA disease characteristics. Numbers in brackets are s.d.

Characteristic	Total (N=287)	cTNC5 positive (N=145)	cTNC5 negative (N=142)	p-value
**Age, years**		60 (12)	58 (11)	0.252
**Male gender**		68	58	0.084
**Dis. duration, years**		13 (10)	12 (9)	0.189
**Swollen joints**		3.9 (4.7)	3.2 (3.8)	0.212
**Tender joints**		3.4 (4.8)	2.9 (4.4)	0.366
**Patient global (0-10)**		4.5 (2.8)	3.9 (2.5)	0.067
**DAS-28-CRP**		4.1 (1.4)	3.8 (1.3)	0.044
**Sharp score**		21 (23)	17 (22)	0.061
**Erosion score**		5 (8)	4 (8)	0.632
**Anti-CCP, U/ml**		187 (123)	101 (117)	<0.001

Supplementary Figure 1.

Mass spectrometry analysis of citrullinated peptides derived from the FGB domain of Tenascin C (accession number P24821). The MS/MS spectra of tryptic peptides 2031-2041, 2042-2051, 2097-2012, 2143-2151, 2148-2157, 2186-2192, 2186-2197, 2193-2199 and 2186-2198 are shown, where r refers to the citrullinated position, indicating that R2033, R2050, R2098, R2147, R2151, R2187, R2192, R2197 and R2198. The matched fragment ions of the y-type (red) and b-type (blue) are shown. The MS/MS spectrum in the bottom panel indicates matches of predominant fragment ions to the peptide sequence.

Supplementary Figure 2.

Relative quantitation of citrullinated peptide occurrence in FBG samples citrullinated with 2 Units of rPAD2, hPAD2 and hPAD4 for 24 hours. (No peptide TR(+.98)YKLK was detected in the sample citrullinated with hPAD4, however this peptide was detected when citrullinated with 20 U of enzyme, data not shown).

Supplementary Figure 3.

Correlation of anti-cTNC5 IgG levels with IgG levels of other ACPA in RA (A) and pre-RA (B) sera. Numbers in corners are percentage of positivity; numbers in parenthesis are percentage of positivity within the CCP2-positive group. The dotted lines indicate cut-off for positivity.

Supplementary Figure 1

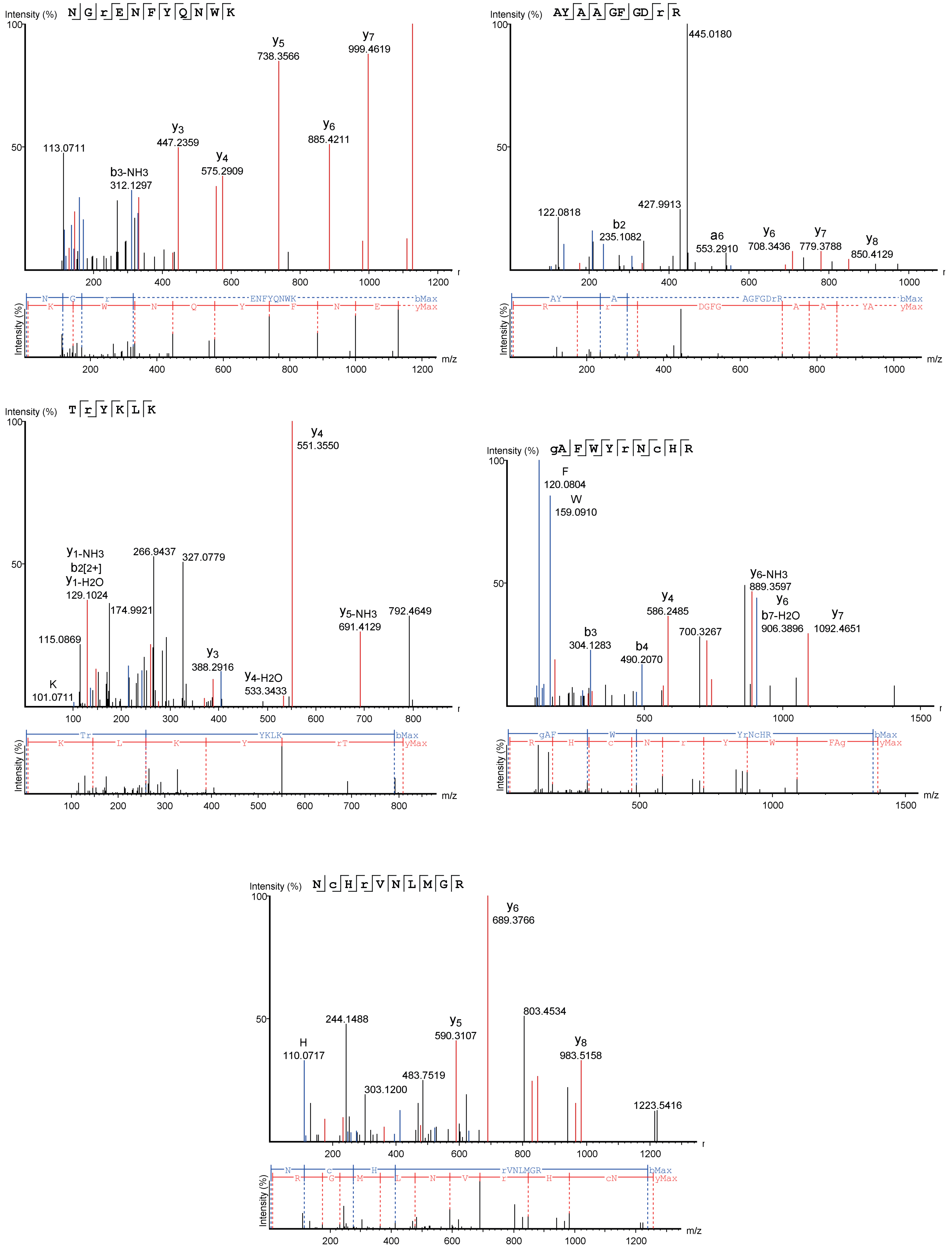

Supplementary Figure 1 - continued

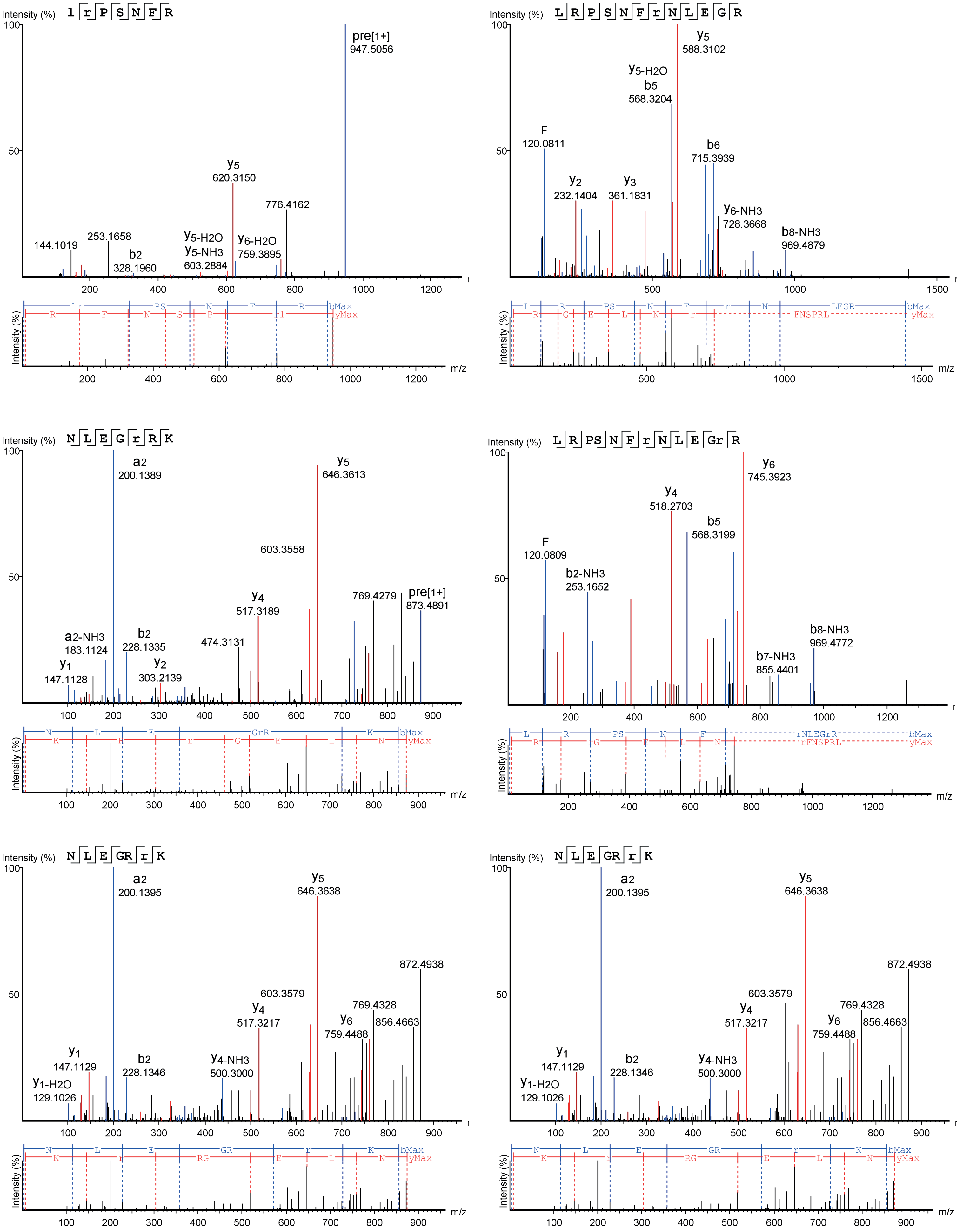

Supplementary Figure 2

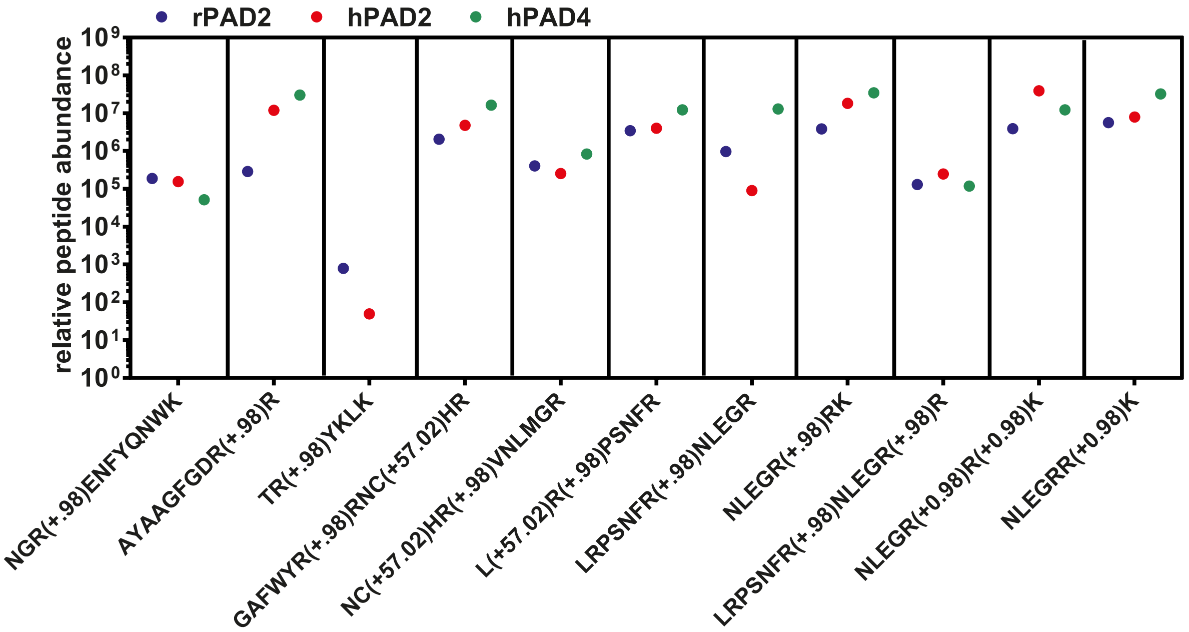

Supplementary Figure 3

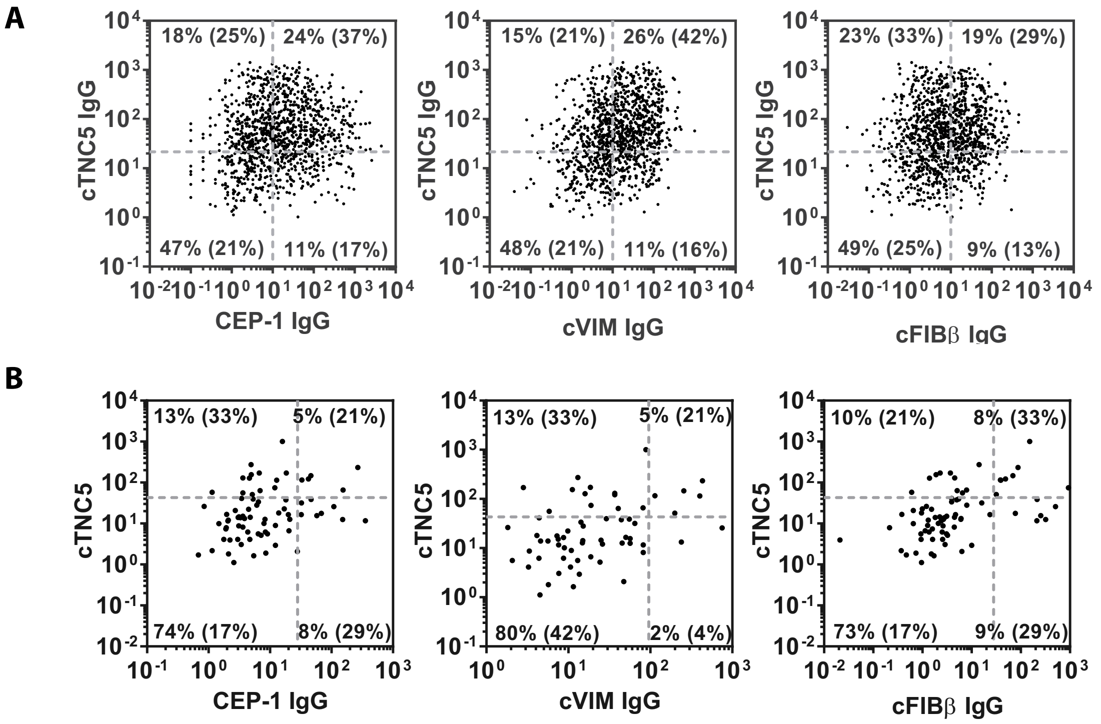

Supplement: Web supplement [file annrheumdis-2015-208495-s1.pdf]
